# Supplementary material for: Prevalence and Variability of Helicobacter pylori Clarithromycin Resistance Mutations in Pediatric Patients in Poland: A Genotypic Analysis Using the Bosphore Genotyping Kit
Source: Antibiotics (Basel). 2025 Mar 31;14(4):352. doi: 10.3390/antibiotics14040352 (PMC12024284; doi:10.3390/antibiotics14040352)
Supplement: Supplementary file 1 [file antibiotics-14-00352-s001.zip › antibiotics-3534140-supplementary.pdf]

Table S1. The detailed distribution of mutation types in the tested samples (*n* = 45).

Patient No.	Result/mutation
1	A2143G
2	WT
3	A2143G
4	A2143G
5	WT
6	WT
7	Invalid (twice)
8	WT
9	WT
10	A2143G
11	WT
12	A2143G and A2142G
13	A2143G
14	WT
15	A2142G
16	WT
17	A2142G
18	WT
19	WT
20	WT
21	Negative
22	A2143G
23	WT
24	WT
25	WT
26	WT
27	WT
28	WT
29	WT
30	WT
31	WT
32	WT
33	WT
34	WT
35	A2143G
36	WT
37	WT
38	A2143G and WT
39	WT
40	WT
41	WT
42	WT
43	WT
44	Negative
45	A2142G

A2142G - adenine to guanine point mutations at position 2142; A2143G - adenine to guanine point mutations at position 2143; WT – wild type.
